# Poor implementation of the EU clinical trial regulation is a major threat for pragmatic trials in European countries

**DOI:** 10.1017/S2045796020000396

**Published:** 2020-05-06

**Authors:** Giovanni Ostuzzi, Chiara Gastaldon, Carlo Petrini, Brian Godman, Corrado Barbui

**Affiliations:** 1 Department of Neuroscience, WHO Collaborating Centre for Research and Training in Mental Health and Service Evaluation, Biomedicine and Movement Sciences, Section of Psychiatry, University of Verona, Verona, Italy; 2 Bioethics Unit, Istituto Superiore di Sanità (Italian National Institute of Health), Rome, Italy; 3 Strathclyde Institute of Pharmacy and Biomedical Sciences, Strathclyde University, Glasgow, UK; 4 Health Economics Centre, Liverpool University Management School, Liverpool, UK; 5 Department of Laboratory Medicine, Division of Clinical Pharmacology, Karolinska Institute, Karolinska University Hospital Huddinge, Stockholm, Sweden

**Keywords:** Antidepressants, psychopharmacology, randomised controlled trials, research design and methods, research governance

## Abstract

The principle of pragmatism in clinical trials has been broadly recognised as a way to close the gap between research and practice. In this contribution, we argue that the conduct of pragmatic clinical trials in Europe may be hampered by poor implementation of current European Union's Clinical Trial Regulation No. 536/2014.

## Introduction

In recent years, the value of pragmatism in clinical trials has been strongly emphasised. Pragmatic trials aim to assess interventions under real-life circumstances by (a) recruiting participants similar to those who would receive the intervention in ordinary care; (b) involving unselected health care facilities; (c) comparing novel interventions with current standards of care; and (d) employing clinically relevant outcomes (Ford and Norrie, 2016). However, as pragmatic designs should resemble clinical practice as much as possible, they might conflict with technical and administrative procedures required by the European Union (EU) for the conduct of experimental studies, including pharmacovigilance procedures. In particular, local implementation of Directive 2001/20/EC, originally ‘[*…*] *aimed at harmonizing clinical trial procedures and improving collaboration among EU member states* [*…*]’, raised a number of administrative and economic issues (The European Parliament and the Council of the European Union, 2001). Consequently, applications for clinical trials in the EU progressively decreased (European Commission, 2012), until a new regulation, called Clinical Trial Regulation No. 536/2014, was issued (The European Parliament and the Council of the European Union, 2014). Novel aspects of this regulation include harmonised electronic submission and assessment processes between EU member states; faster and centralised submission and assessment of trials' protocols; improved information sharing and decision making between and within EU member states; economic facilitations (e.g. concept of co-sponsorship); highest safety standards for patients; increased transparency of information on clinical trials (Gokhale and Berry, 2018; Tenti *et al*., 2018; Scavone *et al*., 2019). Proper implementation of this new regulation remains a challenging process, which actively involves national and local authorities, who are supposed to remove out-to-date, country-specific requirements for the conduction of clinical trials (Gokhale and Berry, 2018). Legislative discrepancies among EU member states is postponing the full implementation of this regulation, although clear data on the situation in each European country are not available (Scavone *et al*., 2019).

In this contribution we argue that poor implementation of the EU regulation is a major barrier towards the development and conduct of pragmatic trials in Europe. We briefly describe a pragmatic clinical trial on antidepressants currently ongoing in Italy, as this represents a case-example of the practical consequences of a lacking implementation of the last EU regulation.

## The VESPA study

The VESPA study (Vortioxetine in the Elderly *v.* SSRIs: a Pragmatic Assessment) is a randomised, open-label, phase IV, multicentre study, involving 13 centres in Italy. The study, financially supported by the Italian Medicine Agency, is currently ongoing, and its protocol is publicly available (ClinicalTrials.gov Identifier: NCT03779789). According to a pragmatic approach, the study will (a) recruit elderly participants with broad inclusion criteria in real-world settings (including both out- and in-patient settings); (b) randomise participants to vortioxetine or one of the selective serotonin reuptake inhibitors (SSRIs) (including sertraline, citalopram, escitalopram, fluoxetine, fluvoxamine, paroxetine), allowing clinicians to choose which SSRI better adapts to each individual participant; and (c) allow the same doctors to simultaneously collect socio-demographic and clinical data, randomise, and prescribe the antidepressant within a reasonable timeframe.

## Concerns about pragmatism

As a result of poor implementation of the EU Clinical Trial Regulation No. 536/2014 in Italy requirements of the previous regulation are still in place and need to be followed. Consequently, participants involved in clinical trials cannot simply get the prescribed medicines at any local community pharmacy, as they would usually do outside an experimental design and as recommended by the new EU regulation (No. 536/214). On the contrary, in experimental settings in Italy, they must receive medicine boxes re-labelled by the pharmacy of the health care facility where the enrolment takes place. Information reported in this additional label (EudraCT code of the study; patient's unique code; name and phone number of the study promoter) is intended to enhance a transparent, easily verifiable and traceable system for the experimental medicines (The European Parliament and the Council of the European Union, 2010). We note that this procedure is associated with at least two feasibility issues that inevitably affect pragmatism.

First, having drugs provided just by the pharmacy of the enrolment site's health care facility consequently reduces the possibility to prescribe medicines to a smaller range, as compared to what is available in the market, as health care facility pharmacies have their own pharmaceutical formularies, which usually include only a selection of medicines available in the community (Bjorkhem-Bergman *et al.*, 2013). In the case of the VESPA study, vortioxetine, although currently marketed in Italy and available in community pharmacies, is not yet available in most health care facility pharmacies. As a consequence, in order to prescribe vortioxetine to participants, additional study funding is needed to purchase it, although in ordinary practice it would be refunded by the National Health System. Furthermore, as health care facility pharmacies usually have only a selection of the SSRIs available, and this selection might differ according to the recruiting centre, the doctor's choice is relevantly limited and much different from usual care, with also a risk of unbalances between centres having different SSRI selections. These limitations, related to regulatory requirements, deeply alter ordinary practice, and are hardly compatible with the original design of the study.

Second, referring the included patients to the health care facility pharmacies, rather than to the local community pharmacies, carries practical issues. Health care facility pharmacies have limited opening hours, which might be a problem both for younger working patients and for elderly participants, particularly those with limited autonomy and needing continuous care. Also, considering the complex therapeutic regimens of most of these patients, they (and their caregivers) might find difficult to collect different medicines at different pharmacies. In order to overcome this challenge, investigators may directly provide re-labelled medicines to the study participants. Although this would ease participants' routine, it still carries practical and methodological limitations. Doctors involved in the recruitment will need a stock of re-labelled medicine boxes in their visiting rooms, and this requires time-consuming procedures to ensure security and regular re-supply. Also, for doctors recruiting patients admitted to medical wards it might be difficult to carry around a whole stock of medication boxes. Moreover, directly providing medicine boxes to participants is likely to affect treatment adherence as compared to everyday practice, where issuing a prescription does not guarantee acquisition and consumption (Blaschke *et al.*, 2012).

**Fig. 1.**
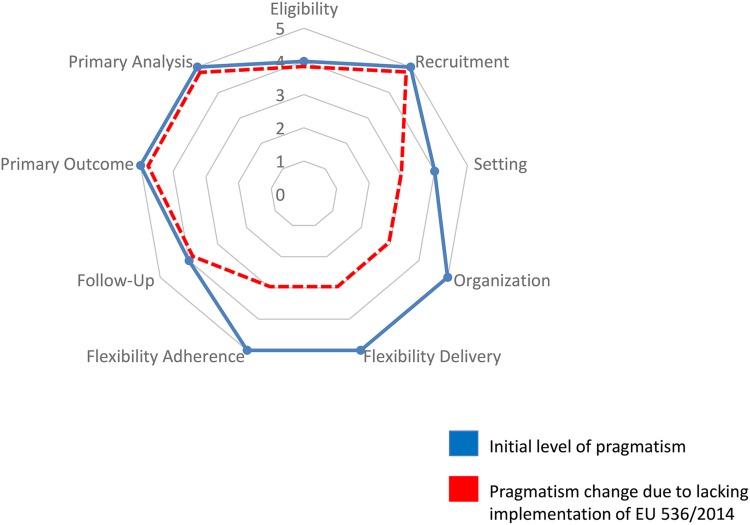
Changes in PRECIS-2 scores after applying EU regulations on pharmacovigilance (higher scores indicate higher levels of pragmatism).

The above-described scenarios represent significant departures from everyday clinical practice, making the recruitment process demanding for both investigators and participants, and profoundly affecting pragmatism. Of notice, this might discourage doctors from community-based non-academic services to take part in the study. The pragmatic value of routinely involving non-academic professionals in clinical trials has been highlighted (Rahman *et al.*, 2011), as they represent the best access point to real-world practice. Figure 1 shows the original level of pragmatism of the VESPA study according to the PRECIS-2 tool (Loudon *et al.*, 2015), and how it is decreased in four different areas (namely flexibility adherence, flexibility delivery, organisation and setting) as a consequence of the current implementation of EU regulations in Italy.

## Future perspectives

The EU Clinical Trial Regulation No. 536/2014 states that ‘*Where the investigational or auxiliary medicinal product have already been placed on the market* [*…*], *as a general rule no additional labelling should be required for clinical trials that do not involve the blinding of the label*’. This simple statement should allow phase IV clinical trials to undergo the same level of pharmacovigilance monitoring applied to ordinary practice. However, the example-case described here shows how this regulation still waits to be properly implemented in Italy. Arguably, similar concerns are to be extended to many other EU state members, although clear data are lacking (Scavone *et al*., 2019). Going further, considering that (a) the level of monitoring in clinical trials is higher compared with clinical practice, and (b) pragmatism enhances the generalisability of study results to real-world populations, one might even argue that highly pragmatic studies represent the best possible source of data for effective pharmacovigilance.

In conclusion, efforts by national (such as the Italian Medicines Agency) and local authorities are urgently required to fully implement the Clinical Trial Regulation No. 536/2014, not only to harmonise trials' procedures, reduce costs, and increase transparency and safety standards, but also to support the conduction of more pragmatic phase IV trials, which represent a seminal step towards data easily generalisable to real-world practice, and, ultimately, better standards of care.
